# The immunological footprint of CMV in HIV-1 patients stable on long-term ART

**DOI:** 10.1186/s12979-015-0041-0

**Published:** 2015-10-01

**Authors:** Jacquita S. Affandi, Jacinta Montgomery, Samantha J. Brunt, David Nolan, Patricia Price

**Affiliations:** School of Pathology and Laboratory Medicine, University of Western Australia, Nedlands, WA Australia; School of Biomedical Science, Curtin University, GPO Box U1987 Bentley, Perth, WA Australia; Institute for Immunology and Infectious Diseases, Murdoch University, Murdoch, WA Australia

**Keywords:** HIV, CMV, ART, Immunosenescence, Age

## Abstract

**Background:**

Most HIV-infected persons are cytomegalovirus (CMV) seropositive and retain latent virus that can be reactivated by immune activation. Their T cell populations express markers reflecting a late stage of differentiation, but the contributions of HIV and CMV to this profile are unclear. We investigated the immunological “footprint” of CMV in HIV patients who had a history of extreme immunodeficiency but were now stable on antiretroviral therapy (ART).

**Results:**

Twenty CMV seropositive HIV patients >50 years old with nadir CD4 T-cell counts <200 cells/μl were studied after >12 years on ART. 16 CMV seropositive and 9 CMV seronegative healthy controls were included. CMV antibody titres were higher in HIV patients than controls (*P* < 0.001-0.003). Levels of soluble B-cell activating factor (sBAFF) were elevated in patients (*P* = 0.002) and correlated with levels of CMV antibodies (*P* = 0.03-0.002), with no clear relationship in controls. CD8 T-cell IFNγ responses to the IE1 peptide (VLE) remained elevated in HIV patients (*P* = 0.005). The CD57^+^CD45RA^+^CD27^−^ phenotype of CD8 T-cells correlated with age (*r* = 0.60, *P* = 0.006), antibodies against CMV IE1 protein (*r* = 0.44, *P* = 0.06) and CD4 T-cell IFNγ response to CMV lysate (*r* = 0.45, *P* = 0.05).

**Conclusions:**

Humoral and T-cell responses to CMV remained elevated in HIV patients after >12 years on ART. Age and presence of CMV disease influenced CD8 T-cell phenotypes. Elevated levels of sBAFF may be a consequence of HIV disease and contribute to high titres of CMV antibody.

**Electronic supplementary material:**

The online version of this article (doi:10.1186/s12979-015-0041-0) contains supplementary material, which is available to authorized users.

## Background

Cytomegalovirus (CMV) infections may be asymptomatic or cause mild symptoms in immunocompetent hosts, but can cause morbidity and mortality in human immunodeficiency virus -1 (HIV) patients. CMV end-organ disease is common in patients with low CD4-T-cell counts, but long term consequences are less clear. At any time, immune activation may promote the reactivation of CMV leading to the re-stimulation of CMV-specific T-cells [1]. This creates T-cell populations enriched with differentiated, apoptosis-resistant memory T cells with limited proliferative capabilities, and leaves an immune system with limited capacity to recognize novel antigens [2]. In the elderly people not infected with HIV, CMV infection has been linked with accelerated immune ageing and/or immunosenescence [3–5], with increased risk for mortality and age-related morbidities [2].

It is reasonable to hypothesize that CMV and other coinfections may contribute to the “accelerated ageing” syndrome observed in HIV-infected individuals [1, 6]. CMV coinfection has been associated with an increased risk of severe non-acquired immune deficiency syndrome (AIDS)-defining events in HIV-infected patients [7]. Untreated HIV infection and chronological ageing are similarly associated with many T cell abnormalities [8, 9]. This includes low CD4/CD8 ratios, low naïve/memory T cell ratios, reduced T cell repertoire, and an expansion of CD57^+^ T cells. CD57 expression can be used to monitor proliferative history, poor proliferative capacity [9], replicative senescence and antigen-induced apoptotic death [10]. Memory T-cells that have undergone multiple rounds of restimulation can also be characterized phenotypically by re-expression of CD45RA [11] and the absence of CD27 [12]. Persistent viral infections, inflammatory syndromes and ageing induce the accumulation of highly differentiated memory T cells re-expressing CD45RA [13]. In HIV infected children and CMV-seropositive healthy children [14], the frequency of CD45RA^+^CD27^−^ phenotype on CD8 T cells correlated with previous CMV infection as measured by serum immunoglobulin G (IgG) levels against CMV. Here we address which CD4 and CD8 T cell markers best define the T-cell phenotype associated with a high burden of CMV in older HIV patients stable on combination antiretroviral therapy (ART).

HIV-seronegative, CMV-seropositive individuals who control CMV replication have very high frequencies of CMV-specific CD8 T-cells able to respond to multiple CMV proteins [15]. Proportions of CMV reactive CD8 T-cells rise rapidly with age in HIV infected patients [16]. The CMV proteins pp65, glycoprotein B (gB) and Immediate Early-1 (IE1) [17] are targets of the CD8 T-cell response against CMV. The peptides NLVPMVATV [“NLV” from CMV pp65] and VLEETSVML [“VLE” from CMV IE1] evoke easily measurable CD8-T-cell responses in healthy CMV-seropositive individuals carrying human leucocyte antigen (HLA)-A*02 [18]. Stone et al. showed that responses to VLE were elevated in previously immunodeficient HIV patients stable on ART when compared to controls [19]. IE1 is expressed early during CMV replication so these cells may be important for protection against CMV reactivation from latency.

Levels of monocyte and B-cell activation are elevated in untreated HIV patients and may remain high on ART [20]. Markers of monocyte activation and tumour necrosis factor (TNF) activity include soluble TNF receptor 1 (sTNFR1) [21] and soluble CD14 (sCD14) [22]. B cell activation can be gauged through levels of total IgG and soluble B-cell activating factor (sBAFF) [23]. BAFF is a novel member of the TNF ligand family and plays an important role in B lymphocyte maturation and survival. BAFF is involved in the pathogenesis of several autoimmune disorders [24] and with risk of long-term kidney graft dysfunction [25].

With the increased availability of ART worldwide, large numbers of patients begin ART with advanced HIV disease and live for many years. This present study investigates T-cell changes as a “footprint” of CMV in a unique cohort of older HIV patients who began ART with advanced immunodeficiency more than twelve years previously and have maintained viral suppression for more than a year. We measured levels of antibodies to CMV lysate, CMV IE1 and CMV gB, using extensive titrations to ensure quantitation in the high range. As titres of all three antibodies were elevated in the HIV patients, we sought explanations for the increase. This included the assessment of sTNFR1, sCD14, total IgG and sBAFF, as well as host interferon gamma (IFNγ) responses of CD4 and CD8 T-cells to CMV proteins.

## Results

### CMV lysate, CMV gB and CMV IE1 antibody titres are higher in HIV patients than healthy controls and more tightly correlated

The study focuses on the long term outcome for CMV seropositive HIV patients stable on ART. HIV patients presented with a median nadir CD4 T-cell count of 78 cells/μL (range: 0–195). Eleven of the 20 HIV patients and 9 out of 16 CMV seropositive (CMV+) controls carried the HLA-A^*^02 allele, but no CMV seronegative (CMV-) controls were carriers. HIV patients had been on ART for more than 12 years [174 (159–189) months] with a median CD4 T-cell count of 691 cells/μL (range: 372–1848) at time of testing.

HIV patients and CMV+ controls were similar in age, but CMV- controls were slightly younger (Table 1). Levels of antibodies reactive with CMV lysate, CMV gB and CMV IE1 were higher in HIV patients than CMV+ controls (Table 1). In HIV patients, levels of antibodies reactive with different CMV antigens were tightly correlated (lysate *vs* gB, *r* = 0.81, *P* < 0.0001; lysate *vs* IE1, *r* = 0.70, *P* = 0.0005; gB *vs* IE1, *r* = 0.57, *P* = 0.009). In CMV+ controls, similar trends were evident (lysate *vs* gB, *r* = 0.57, *P* = 0.02; lysate *vs* IE1, *r* = 0.52, *P* = 0.04) although CMV gB and CMV IE1 did not differ (*r* = 0.17, *P* = 0.53).

**Table 1:** HIV patients stable on ART retain elevated humoral and cellular responses to CMV and advanced T-cell senescence

	HIV patients	CMV + Controls	CMV- Controls	*A vs B*	*A vs C*	*B vs C*
	A	B	C	*P* ^b^	*P* ^b^	*P* ^b^
*n*	20	16	9			
Male:Female	19:1	14:2	9:0			
Age (years)	62.5 (50–73)^a^	60 (50–74)^a^	55(52–69)^a^	0.19	**0.03**	0.12
Levels in Plasma						
CMV lysate antibody AU/L	94 (23–995)	20 (6–83)	0.8 (0.5–1.1)	**<0.0001**	**<0.0001**	**<0.0001**
CMV gB antibody AU/L	127 (27–400)	45 (2–88)	2 (0.6–3.3)	**<0.0001**	**<0.0001**	**<0.0001**
CMV IE-1 antibody AU/L	49 (8–1098)	9 (2–180)	2.6 (1.9–10)	**<0.0001**	**<0.0001**	**0.003**
sCD14 ng/mL	22 (7.7–48)	18 (8.3–39)	22 (11–31)	0.09	0.23	0.68
sTNFR1 pg/mL	15 (9.1–25)	15 (12–27)	17 (10–21)	0.68	0.83	0.72
Total IgG mg/mL	12 (3–22)	12 (5–20)	9.4 (6.5–15)	0.46	0.16	0.14
sBAFF ng/mL	740 (376–1401)	519 (274–786)	319 (318–471)	**0.002**	**0.0003**	*0.06*
IFNγ spots per 2 × 10^6^ cells						
CMV lysate	227 (16–700)	157 (13–617)	0 (0–0.5)	0.16	**<0.0001**	**<0.0001**
CMV pp65	445 (14–1591)	138 (18–645)	0 (0–2)	**0.005**	**<0.0001**	**<0.0001**
CEF control peptide pool	516 (2–1500)	337 (20–584)	4 (0–404)	*0.07*	**0.001**	**0.0015**
NLV peptide	498 (56–1363)^c^	214 (13–651)^d^	na	*0.06*	na	na
VLE peptide	420 (14–2000)^c^	25 (7–561)^d^	na	**0.005**	na	na
T cell subset [as % of]						
CD4^+^ T cells [lymphocytes]	43 (24–77)	69 (52–84)	69 (52–80)	**<0.0001**	**0.0009**	0.93
CD57^+^ [CD4]	11 (2–75)	8 (2–26)	4.5 (1.7–7.4)	0.11	**0.001**	**0.03**
CD57^+^CD45RA^+^CD27^−^ [CD4]	1.9 (0–57)	0.44 (0.06–14)	0.02 (0.005–0.16)	*0.06*	**0.0002**	**0.0001**
CD8^+^ T cells [lymphocytes]	48 (16–71)	22 (7–43)	21 (15–44)	**0.0001**	**0.002**	0.97
CD57^+^ [CD8]	47 (17–67)	40 (6.4–69)	28 (10–68)	*0.08*	**0.04**	0.51
CD57^+^CD45RA^+^CD27^−^ [CD8]	19 (4.2–53)	26 (4–49)	8 (2–19)	0.61	**0.03**	**0.0001**

*na* = Not applicable as none of the CMV-seronegative healthy controls carried the HLA-A*02 allele

^a^Median (range)

^b^Mann–Whitney, *P* ≤ 0.05 (**bold**), *P* > 0.05-0.1 (*italics*)

^c^HLA-A*02 allele restricted thus HIV+ patients *n* = 11

^d^CMV+ controls *n* = 9

High CMV antibody levels in HIV patients may reflect increased exposure to CMV antigens before they began ART, but could also indicate persistent B-cell activation. Hence, we assessed levels of sBAFF and total IgG to examine whether antibody levels reactive with CMV reflect polyclonal B-cell activation.

### B-cell activation (sBAFF and IgG), but not monocyte activation (sCD14 and sTNFR1), may contribute to high CMV antibody titres in HIV patients

HIV patients had higher levels of sBAFF than CMV+ controls (Table 1). There was a direct relationship between CMV antibodies and sBAFF in patients [CMV lysate (*r* = 0.72, *P* = 0.002), CMV gB (*r* = 0.70, *P* = 0.003), CMV IE1 (*r* = 0.54, *P* = 0.03)]. However in CMV+ controls, a weak inverse relationship was observed between levels of sBAFF and CMV lysate (*r* = −0.47, *P* = 0.08) and CMV IE1 (*r* = −0.51, *P* = 0.06)]. In HIV patients, sBAFF levels correlated with levels of total IgG (*r* = 0.70, *P* = 0.003), but this was not seen in CMV+ controls (*r* = −0.53, *P* = 0.04).

Levels of total IgG in HIV patients correlated with antibodies to CMV gB (*r* = 0.65, *P* = 0.002) and CMV lysate (*r* = 0.40, *P* = 0.08) however these observations were not evident in CMV+ controls (*r* = 0.09 to 0.30, *P* = 0.26 to 0.74).

Levels of sCD14 and sTNFR1 were similar in all groups (Table 1). In CMV+ controls, sCD14 levels correlated inversely with CMV antibodies (CMV lysate, *r* = −0.50, *P* = 0.05; CMV gB, *r* = −0.51, *P* = 0.05; CMV IE1, *r* = −0.49, *P* = 0.06), whilst these parameters were unrelated in patients (*r* = 0.04 to 0.35, *P* = 0.13 to 0.86). sTNFR1 levels did not correlate with antibodies to CMV antigens in any group.

### IFNγ responses to the CMV IE1 peptide (VLE) remain elevated in HIV patients

IFNγ responses were assessed by enzyme linked immunosorbent spot assay (ELISpot) in peripheral blood mononuclear cells (PBMC) stimulated with whole CMV lysate (mediated by CD4 T-cells), CMV pp65 peptide pool (mediated by CD4 and CD8 T-cells), CMV, EBV and influenza (CEF) control peptide pool (mediated by CD8 T-cells), and HLA-A*02 restricted CMV peptides (VLE and NLV; mediated by CD8 T-cells) [26].

HIV patients had more CD4 and CD8 T-cells producing IFNγ in response to CMV pp65 peptide pool (*P* = 0.005, Table 1) and more CD8 T-cells producing IFNγ in response to VLE peptide (*P* = 0.005) than CMV+ controls. However, numbers of CD4 T-cells responding to CMV lysate were similar in patients and CMV+ controls (Table 1) and responses to the CEF peptide pool were only marginally lower in CMV+ controls (Table 1). This had been described in patients from our clinic tested after 4 years on ART and 6 months of complete viral suppression [19]. As expected, CMV- controls had low/undetectable IFNγ responses to CMV lysate (Table 1), CMV pp65 peptide pool (Table 1) or even CEF control peptide pool (*P* = 0.001) (Table 1).

### In HIV patients, expression of CD57^+^CD45RA^+^CD27^−^ on CD8 T-cells correlated with age, CMV IE1 and CD4 T-cell IFNγ responses to CMV lysate

Proportions of CD8 T-cells in HIV patients were higher than in controls (Table 1), but the CD57^+^ or CD57^+^CD45RA^+^CD27^−^ phenotypes of CD8 T-cells were equally common in HIV patients and CMV+ controls (Table 1). However CMV- controls had few CD8 T-cells expressing CD57^+^CD45RA^+^CD27^−^, so the accumulation of these cells is driven by CMV.

In HIV patients, proportions of CD8 T-cells with highly differentiated phenotypes (CD57^+^ or CD57^+^CD45RA^+^CD27^−^) correlated with age (*r* = 0.67, *P* = 0.001; *r* = 0.60, *P* = 0.006, resp), antibodies reactive with CMV IE1 (*r* = 0.44, *P* = 0.06; *r* = 0.41, *P* = 0.07) and IFNγ responses to CMV lysate (*r* = 0.45, *P* = 0.05; *r* = 0.42, *P* = 0.07). Expression of CD57^+^ on CD8 T-cells was also correlated with IFNγ responses to the CMV IE1 VLE peptide (*n* = 9; *r* = 0.60, *P* = 0.05) and age (*r* = 0.67, *P* = 0.001). CMV+ controls did not show equivalent correlations.

HIV patients retained lower proportions of CD4 T-cells than CMV+ or CMV- controls (Table 1). Proportions of CD4 T-cells with the phenotype CD57^+^CD45RA^+^CD27^−^ were marginally higher in HIV patients than in CMV+ controls (Table 1). CMV+ controls had more CD57^+^ or CD57^+^CD45RA^+^CD27^−^ CD4 T-cells than CMV- controls so again CMV is the driving force for CD4 T-cell differentiation (Table 1). Accordingly in patients, CD57 expression on CD4 T-cells correlated weakly with antibody and IFNγ responses to CMV lysate (*r* = 0.41, *P* = 0.07; *r* = 0.39, *P* = 0.09).

## Discussion

CMV seropositivity has been placed in an “immune risk profile” associated with mortality in longitudinal studies of people over 85 years old [3, 5]. Here, we describe investigations of a unique cohort of CMV seropositive HIV patients over 50 years of age who began ART with advanced immune deficiency over 12 years earlier and maintained undetectable plasma HIV RNA for more than a year. Their levels of antibodies reactive with CMV lysate, gB and IE1 remained elevated despite the long period on effective ART.

Elevated sBAFF levels have been associated with autoimmune diseases [27], graft versus host disease [28], and complications of kidney transplantation [29]. In CMV-deoxyribonucleic acid (DNA) positive renal transplant patients, sBAFF levels were higher than in CMV-DNA negative recipients, with positive correlations between CMV-DNA levels, total IgG and sBAFF [30]. Here, levels of sBAFF and total IgG correlated with antibodies reactive with CMV lysate, CMV gB and CMV IE1 in HIV patients. This suggests that B-cell activation is a feature of HIV disease and contributes to elevated titres of CMV antibodies. Although we found no associations between CMV antibodies and levels of sTNFR1 or sCD14, this may be apparent in extended studies that include HIV+CMV- patients and assess CMV-DNA. HIV+CMV- patients are rare so collaborative studies will be needed.

HIV+CMV+ patients had higher frequencies of CD8 T-cells specific for the CMV IE1 VLE peptide than CMV+ healthy controls, whilst frequencies of CD8 T-cells specific for the CMV pp65 NLV peptide were less clearly elevated, as expected [19]. IE1 proteins are expressed before pp65 during CMV reactivation, thus HIV patients on ART may experience CMV reactivation more frequently than controls. IFNγ responses to CMV pp65 peptide pool and CMV IE1 protein were also elevated in subjects >85 years old not infected with HIV [31]. No HIV patients displayed symptoms of CMV disease at the time PBMC were collected, so VLE-specific CD8 T-cells may play a role in averting CMV replication.

Elevated CD8 T-cell IFNγ responses to VLE may reflect oligoclonal expansion of CMV-specific CD8 T-cells before ART, as many patients in this study had a history of CMV end-organ disease. Long-term ART does not increase the diversity of T-cell repertoires in HIV patients despite sustained suppression of viral replication [32].

As CMV seropositivity has been linked to “accelerated ageing” [1], highly differentiated effector memory CD4 and CD8 T-cells were assessed by expression of CD57 and CD45RA without CD27 [12]. Proportions of CD8 T-cells with the phenotype CD57^+^CD45RA^+^CD27^−^ correlated with age and with antibodies against the CMV IE1 protein and CD4 T cell IFNγ response to CMV lysate. Thus demonstrates that CMV seropositivity may accelerate CD8 T-cell differentiation and further worsen the impairment caused by HIV.

HIV patients had marginally more highly differentiated CD4 T-cells than CMV+ controls. Proportions of CD4 and CD8 T-cells from HIV patients expressing CD57 correlated with IFNγ responses to CMV antigen and peptide (respectively), consistent with CD57^+^ T-cells selectively retaining the capacity to produce IFNγ [26].

## Conclusions

Overall these results establish that the immunological “footprint" of CMV remains elevated in HIV patients after more than 12 years on ART and correlates with CD8 T-cell phenotypes. Elevated levels of sBAFF may be an effect of HIV and contribute to high titres of CMV antibody. Extension of our study in larger cohorts should include CMV-seronegative HIV patients and CMV-DNA assessments to further elucidate the mechanisms by which CMV reactivation accelerates immune ageing in HIV patients.

## Methods

### Patient and control cohorts

Twenty CMV-seropositive HIV patients were selected from the HIV database of the Department of Clinical Immunology and Immunogenetics, Royal Perth Hospital, Western Australia. Participants were all >50 years old with nadir CD4 T-cell counts < 200 cells/ul, studied after >12 years on ART and >1 year of complete viral suppression (<50 HIV RNA copies/ml). All participants were healthy at the time of sample donation. ART comprised at least three antiretroviral drugs including a non-nucleoside reverse transcriptase inhibitor or protease inhibitor. Sixteen CMV-seropositive healthy controls (designated CMV+ controls) and nine CMV-seronegative healthy controls (designated CMV- controls) were included. Seropositivity was defined as >1100 AU/mL of CMV lysate antibody, where the cut-off was based on eleven samples from persons confirmed to be CMV seronegative by routine serology (Abbott Diagnostic Systems, Lake Forrest, IL, USA). Informed consent was obtained from all participants, and the human experimentation guidelines of Royal Perth Hospital and University of Western Australia were followed. Samples were screened for carriage of HLA-A^*^02 by flow cytometry [19]. Eleven of 20 (55 %) patients, nine of 16 (56 %) CMV+ controls and no CMV- controls, carried HLA-A^*^02.

### T-cell subset phenotyping

Freshly collected PBMC [isolated by ficoll density gradient centrifugation (Ficoll-Paque Plus; GE Healthcare Biosciences, Sweden)] were stained for surface markers (15 min, room temperature) using conjugated monoclonal antibodies as follows: CD8-FITC (clone SK1), HLA-DR-PE (clone TU36), CD57-APC (clone NK-1), CD45RA-APC-H7 (clone HI100), CD27-PerCPCy5.5 (clone M-T271), CD3-V450 (clone UCHT1) and CD4-V500 (clone RPA-TA) from BD Biosciences (San Jose, CA, USA). Stained cells were washed twice and analysed on a FACSCanto^TM^ (BD Biosciences). At least 200 000 events were acquired and analysed using FlowJo (Treestar, San Carlos, CA, USA). Expression of CD57, CD27 and CD45RA was used to define terminally differentiated effector memory (T_EMRA_) (CD57^+^CD45RA^+^CD27^−^) T-cells. For comparisons with other studies, we also identified senescent T-cells solely by expression of CD57. Gating strategies can be found in Additional file 1: Figure S1.

### Immune activation and total IgG ELISA

Levels of sTNFR1, sCD14 (RnD Systems; Minneapolis, MI, USA) and sBAFF (Abcam; Cambridge, UK) in plasma were quantified using commercial reagents. Plasma was serially diluted threefold from 1:9 for sTNFR1, 1:200 for sCD14 and 1:3 for sBAFF enzyme linked immunosorbent assay (ELISA). Binding of the peroxidase-conjugated secondary antibody was detected with a tetramethylbenzidine (TMB) substrate (Sigma-Aldrich; St Louis, MI, USA). Reactions were stopped with 1 M sulphuric acid (H_2_SO_4_) and quantified at 450 nm. Four-parameter logistic curves were generated from >6 titrations of the standard using SOFT max PRO version 5.4 software.

Total IgG was quantified using plates coated with polyvalent goat anti-human IgG (2.5 mg/mL; Invitrogen; Carlsbad, CA, USA) diluted 1:500 in bicarbonate buffer and blocked with 5 % bovine serum albumin (BSA) in phosphate buffered saline (PBS) for 60 min. Plasma samples were serially diluted threefold from 1:100,000 in 2 % BSA/PBS and applied for 120 min. Binding was detected using goat anti-human IgG conjugated horseradish peroxidase (HRP) (Sigma-Aldrich) followed by TMB substrate as described above. Hypergammaglobulineamia was defined as >14 g/L IgG, as the normal range is 6-14 mg/mL [33].

### CMV-specific antibody ELISAs

IgG reactive with CMV was quantitated using CMV lysate, CMV gB and CMV IE1. CMV lysate was prepared by sonication of human foreskin fibroblasts (HFF) infected with CMV strain AD169. Uninfected HFF were prepared in parallel to create a control lysate. Replicate plates were coated with CMV gB (produced in hamster ovary cells, Chiron Diagnostics, Medfield, MA, USA; 2800–800; 50 ng/mL) or CMV IE1 (produced in *E.coli*, Miltenyi Auburn, CA; 2500 ng/mL). Plasma samples were diluted from 1:200. Bound IgG was detected as described above. A plasma sample assigned a value of 1000 units IgG reactive with each antigen was run on each plate, and unit values were derived for all samples. The units calculated from uninfected fibroblasts were subtracted from those generated using CMV-coated plates.

### Detection of IFN-γ producing T-cells

ELISpot assays utilised anti-IFN-γ antibodies purchased from MabTech (Stockholm, Sweden) and cryopreserved PBMC with cell viability >95 % described elsewhere [26]. Cells were stimulated with anti-CD3 (10 ng/mL; MabTech), whole CMV lysate (described above), CEF control peptide pool [contains peptides from CMV, Epstein-Barr virus (EBV) and Influenza; 2 μg/mL; MabTech), CMV pp65 peptide pool (NIH AIDS reagent program, DAIDS, NIAID and Immunodiagnostics, Woburn, MA, USA), HLA-A^*^02-restricted peptides NLV (residues 495–503 of pp65) or VLE (residues 316–324 of IE-1) at 10 μg/mL (Proteomics International, Perth, Australia). Spots >10 units in size and >20 units in intensity were counted using an AID ELISpot Reader System (AID, Strassberg, Germany). Numbers of spots in unstimulated wells were subtracted from numbers in stimulated wells and adjusted per 2 × 10^6^ PBMC.

### Statistical analysis

Results were presented as median (range) values unless otherwise stated. Bivariate analyses were based on Mann Whitney tests. Correlation coefficients were determined by the Spearman’s Rank Correlation Test (GraphPad Prism version 5, La Jolla, CA, USA). For all tests, *P* < 0.05 were considered to represent a significant difference and highlighted in **bold**, whereas 0.05 < *P* < 1 is *italicized*.

## Additional file

Additional file 1: Figure S1. Senescent T cell gating strategy. Lymphocytes were distinguished from monocytes by their forward and side light scatter (**a**), gated for expression of CD3 (**b**), CD4 and CD8 (**c**). Quadrant gates were then set for expression of CD45RA and CD27 within the CD4^+^ (**d**) and CD8^+^ (**e**) populations. Gating was further set for expression of CD57^+^ within the CD45RA^+^ CD27^−^ CD4^+^ (**f**) and CD45RA^+^ CD27^−^ CD8^+^ (**g**) populations. (PDF 261 kb)

## Abbreviations

AIDS: Acquired immune deficiency syndrome
ART: Antiretroviral therapy
AU: Arbitrary units
BSA: Bovine serum albumin
CD-: Cluster of differentiation
CEF: CMV, EBV and influenza control peptide pool
CMV-: CMV seronegative healthy controls
CMV: Cytomegalovirus
CMV+: CMV seropositive healthy controls
DNA: Deoxyribonucleic acid
EBV: Epstein-Barr virus
ELISA: Enzyme linked immunosorbent assay
ELISpot: Enzyme linked immunosorbent spot assay
gB: Glycoprotein B
H_2_SO_4_: Sulphuric acid
HFF: Human foreskin fibroblasts
HIV: Human immunodeficiency virus-1
HLA-: Human leukocyte antigen
HRP: Horseradish peroxidase
IE1: Immediate early 1 protein
IFNγ: Interferon gamma
IgG: Immunoglobulin G
NLV: NLVPMVATV peptide from CMV pp65 protein
PBMC: Peripheral blood mononuclear cells
PBS: Phosphate buffered saline
pp65: CMV structural protein
sBAFF: Soluble B-cell activating factor
sCD14: Soluble cluster of differentiation 14
sTNFR1: Soluble tumour necrosis factor receptor 1
T_EMRA_: Terminally differentiated effector memory T-cells
TMB: Tetramethylbenzidine
TNF: Tumour necrosis factor
VLE: VLEETSVML peptide from CMV IE1 protein
