# Supplementary figures and images for: The immunological footprint of CMV in HIV-1 patients stable on long-term ART
Source: Immun Ageing. 2015 Oct 1;12:14. doi: 10.1186/s12979-015-0041-0 (PMC4591633; doi:10.1186/s12979-015-0041-0)

Supplementary Figure 1 – Senescent T cell gating strategy

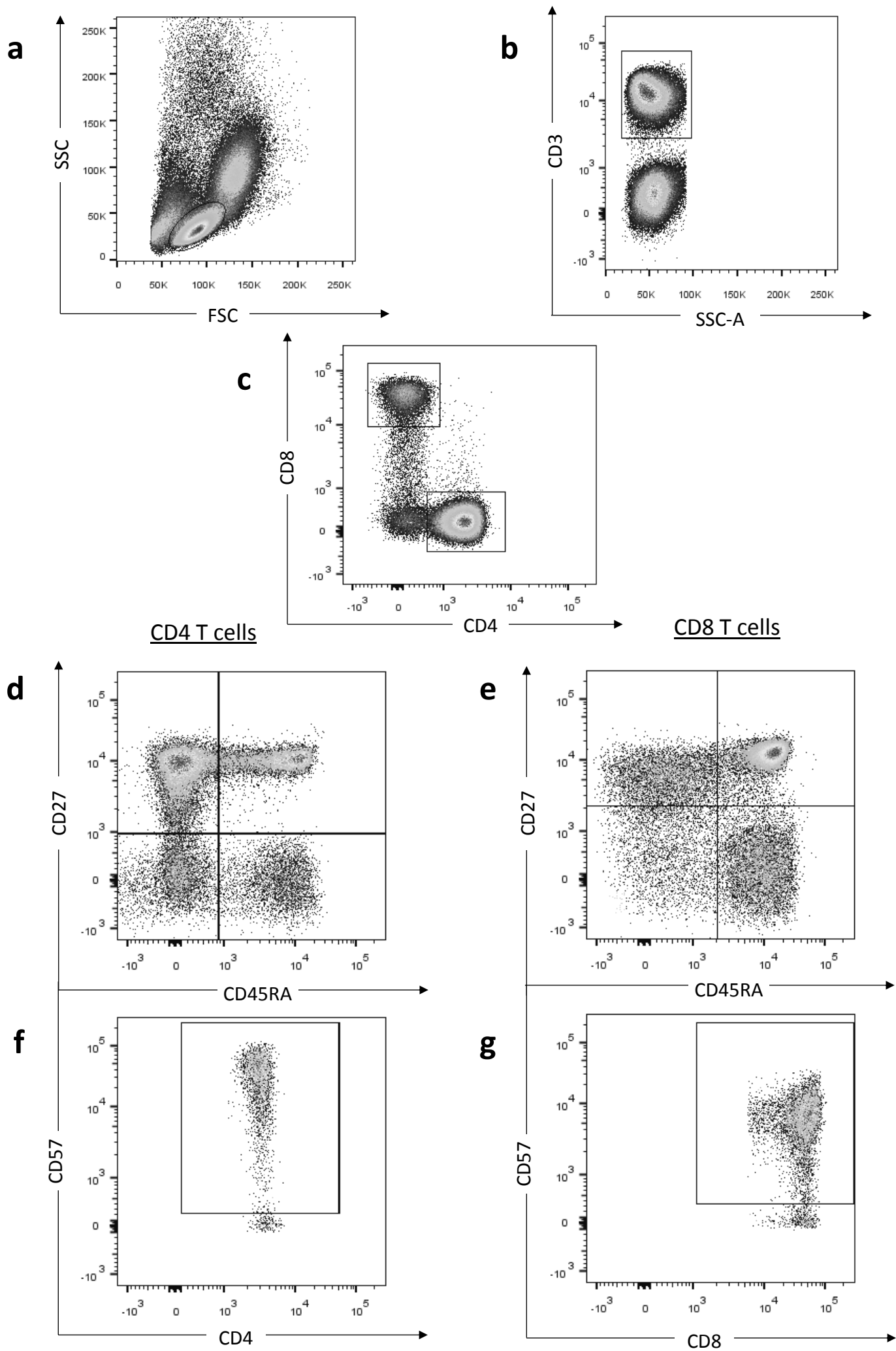

Supplement: Additional file 1: Figure S1. — Senescent T cell gating strategy. Lymphocytes were distinguished from monocytes by their forward and side light scatter (a), gated for expression of CD3 (b), CD4 and CD8 (c). Quadrant gates were then set for expression of CD45RA and CD27 within the CD4+ (d) and CD8+ (e) populations. Gating was further set for expression of CD57+ within the CD45RA+ CD27− CD4+ (f) and CD45RA+ CD27− CD8+ (g) populations. (PDF 261 kb) [file 12979_2015_41_MOESM1_ESM.pdf]
